# Field investigation of non-uniform environment in a Venlo-type greenhouse in Yangling, China

**DOI:** 10.1016/j.heliyon.2023.e22143

**Published:** 2023-11-10

**Authors:** Xianpeng Sun, Jinhong He, Chuanzhen Li, Yangda Chen, Runjie Li, Ziteng Wang, Weijun Wu, Yapeng Li, Xuxin Guo, Xinke Wang

**Affiliations:** a College of Horticulture, North West Agriculture and Forestry University, Yangling 712100, Shaanxi Province, China; b Key Laboratory of Horticultural Engineering in Northwest Facilities, Ministry of Agriculture, Yangling 712100, Shaanxi Province, China; c Facility Agriculture Engineering Technology Research Center of Shaanxi Province, Yangling 712100, Shaanxi Province, China; d School of Human Settlements and Civil Engineering, Xi'an Jiaotong University, Xi'an, 710049, Shaanxi Province, China

**Keywords:** Greenhouse, Indoor environment, Building energy, Horticulture, Air conditioning

## Abstract

Non-uniform environmental conditioning has established substantial energy-saving and conditioning effects in residential buildings, however, few studies on the technology applied in greenhouses have been conducted. Semi-enclosed greenhouse development is hindered by energy consumption. To better apply non-uniform environmental conditioning technology in greenhouses, it is necessary to investigate the non-uniform characteristics of field environment parameters. Therefore, spatial and temporal measurements of indoor temperature and relative humidity in a Venlo-type greenhouse in Yangling, China, were conducted on June 5–11, 2022. Temperature and humidity sensors were arranged in the greenhouse at 4.5 m intervals, in the canopy, cultivation, center, and root areas. Temperature and humidity measurement points on the greenhouse walls were selected. The measurement results showed large fluctuations in the indoor temperature and relative humidity over time. The difference between indoor and outdoor average temperatures ranged from -5–10 °C and temperatures unsuitable for tomato growth were identified, although some passive conditioning methods such as ventilation and water spraying were employed, which indicates the necessity of active heating and cooling. Based on the measured data, the nonuniformity coefficients of temperature and relative humidity in different directions in the greenhouse were calculated. A larger non-uniformity in the vertical direction was found compared to that in the horizontal direction. These results suggest the possibility of non-uniform environmental conditioning. A rough estimation of the energy consumption by the two different condition modes, namely zone-specific and overall conditioning, was made. A huge energy saving of 69.6 % by the zone-specific conditioning mode was revealed compared to the overall conditioning. This implies a huge advantage in energy efficiency by non-unform environmental conditioning technologies applied in greenhouses. The study provides useful data for understanding non-uniform environments in greenhouses and the application of non-uniform environmental conditioning technologies.

## 1 Introduction

The concentration of carbon dioxide (CO_2_) is rising at an alarmingly rapid rate, which has contributed to drastic changes in the global climate [1]. Measures have been implemented to mitigate CO_2_ emissions in many countries [2]. Particularly in China, carbon peak and carbon neutral goals have been established, and many effective policies have been improved with the aim of decreasing CO_2_ emissions. Some studies show that agricultural carbon emissions account for 16–17 % of the total carbon emissions in China, which is higher than the global average of 13.5 %, and is still growing at an average annual rate of 5 % [3]. Therefore, it is important to reduce agricultural carbon emissions to achieve carbon peak and carbon neutral emission targets. With the growth of agriculture in China, facility agriculture is becoming a key issue in boosting agricultural development and an important measure to advance rural revitalization. Facility agriculture is a high-input, high-tech, and high-output industry that consumes considerable energy [4]. In particular, large continuous greenhouses and plant factories require intensive energy to meet high environmental control requirements. The annual energy consumption of environmentally-controllable agriculture is reported to be 17,382.4 kWh, with lighting and HVAC accounting for 69.9 % and 27.9 % of annual energy consumption, respectively [5]. The high energy consumption issue in greenhouses has restricted the development of agricultural facilities and does not meet the national requirements for future development regarding agricultural energy consumption. Reducing greenhouse energy consumption and carbon oxide emissions is an urgent problem that needs to be solved.

There are many ways to achieve energy saving in greenhouses, including three main aspects: (1) to use cleaner and more durable energy at the energy end; (2) to lower energy requirements through more precise and rational distribution of energy at the control end; and (3) to adapt the energy supply to energy requirements in the greenhouse. Among them, the control of greenhouse environmental parameters is crucial for improving yield and energy use efficiency, and the greenhouse environmental control system directly determines the environmental parameters in the greenhouse [6]. Many researchers have already conducted exhaustive studies on the control of environmental parameters in greenhouses including temperature [[6], [7], [8], [9]], humidity [[10], [11], [12]], CO_2_ concentration [13,14], light [[15], [16], [17]] and so forth. However, only a small fraction of the space in most greenhouses is used for crop cultivation, although the vertical farm concept has been developed to improve the space utilization rate. Hence, a large amount of energy is wasted for environmental conditioning in the entire space, whereas it is only necessary in the partial space containing crops. Therefore, non-uniform environmental regulation in large greenhouses may be a good way to lower energy consumption as plants occupy only a small portion of the greenhouse. In fact, non-uniform environmental regulations have been rapidly developing in civil buildings to reduce energy consumption, which refers to regulating the environment in local areas only according to actual demand, rather than the entire environmental space, to achieve energy saving in buildings while meeting demand [18].

Different non-uniform regulation modes have been studied in residential and public buildings, especially in large-space buildings, including displacement [19,20], stratum [21], wall-attached [22], personalized [23] and other demand-controlled ventilation [24]. Although there are different air supply and exhaust designs, all these non-uniform regulation modes aim to control the thermal comfort and indoor air quality around human bodies instead of the entire indoor environment [25]. Owing to small space environmental regulation, the energy consumption obtained from non-uniform environmental regulation is reduced compared to that from mixing environmental conditioning. Numerical analysis showed that displacement and stratum ventilation may save energy by approximately 19 % and 44 %, respectively, over a year, compared to mixing ventilation [26]. A case study in a two-story island metro station showed that wall-attached ventilation can reduce the supply air flow rate of the air handling unit and chiller capacity by 20 % and 22 %, respectively, compared with mixing ventilation [27]. Experimental measurements have revealed that personalized ventilation may reduce energy consumption by up to 51 % [28]. The study confirmed the energy-saving potential and realizability of non-uniform regulation in residential and public buildings. Recently, displacement ventilation has also shown an annual energy cost decrease of approximately 17.5 % less than that for mixing ventilation in machining plants [29]. Despite the large potential for energy saving in applying non-uniform environmental regulations in greenhouses, few studies have been conducted in this field. Ishii et al. [30] measured and simulated temperature distributions in a naturally-ventilated multi-span greenhouse and found obvious temperature non-uniformity in an indoor environment. Lee et al. revealed a temperature difference of up to 3.0 °C and 6.5 °C length- and widthwise, respectively, in a 75 × 8 m greenhouse during a typical winter season [31]. Xu et al. [32] employed computational fluid dynamics to investigate the non-uniform temperature in a single-span greenhouse. However, these studies indicated that even the environment should be improved for crop cultivation, which is true in crop cultivation zones, however, it may not be suitable for environmental regulation in the entire greenhouse. Active non-uniform environmental regulation with coordination between energy saving and healthy crop growth has not received sufficient attention. Prior to that, it was necessary to study the non-uniform environmental field in greenhouses under existing control methods. Therefore, the objectives of this study were to investigate the non-uniformity of the environmental field in the greenhouse under existing regulation methods, focusing on the stratification of environmental parameters in the greenhouse, and qualitatively estimate the energy-saving potential of non-uniform regulation compared with the overall regulation mode with tomato cultivation temperature as the target.

## 2 Methods

### 2.1 Experimental greenhouse

**Fig. 1:**
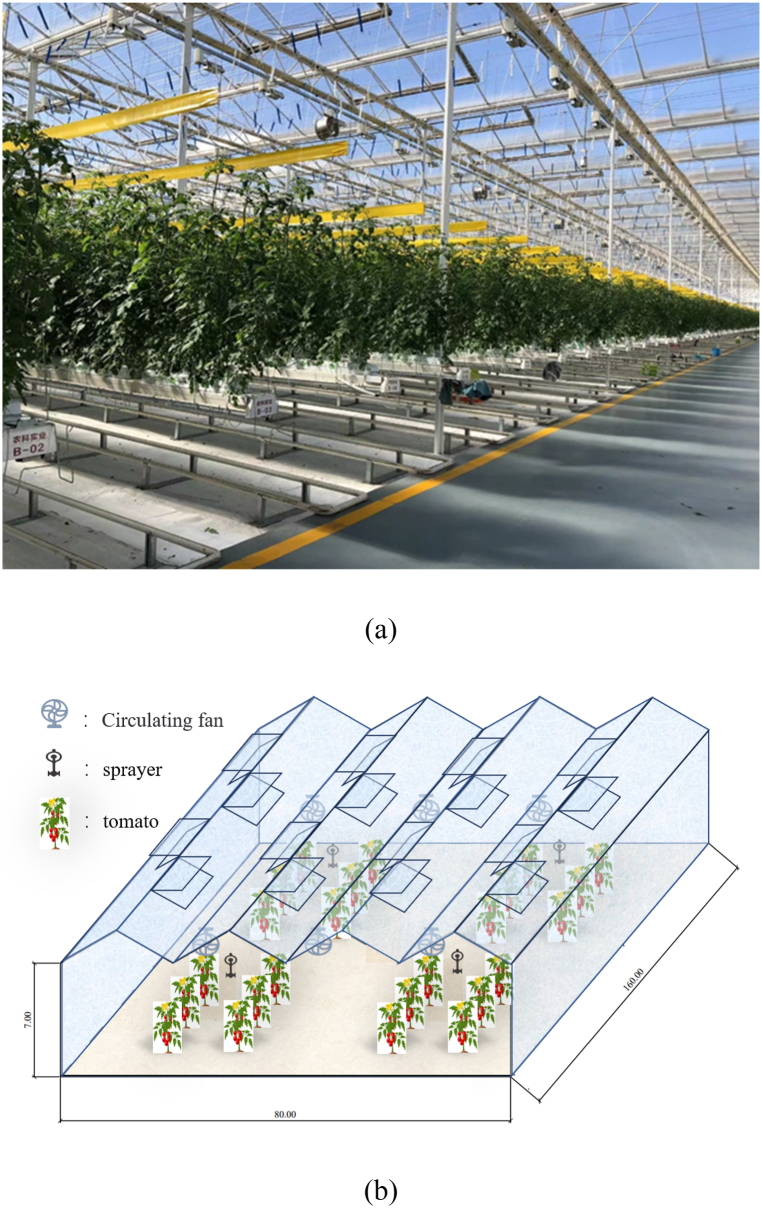
The Venlo-type multi-span greenhouse. (a) the photo of the greenhouse; (b) the schematic show of the greenhouse structure.

The greenhouse used in this experiment was a Venlo-type multi-span glass greenhouse, as shown in Fig. 1(a), located in Yangling Intelligent Agricultural Demonstration Park in Shaanxi Province, with a longitude and latitude of 34°17′ N and 108°03′ E, respectively. The region is flat terrain and has a warm temperate and semi-humid monsoon climate with high ambient temperature of more than 40 °C, strong solar radiation, and low wind speed in summer, cold with ambient air temperature below 0 °C in winter. Therefore, a test greenhouse requires a large amount of energy to regulate the greenhouse environment, regardless of winter or summer. The greenhouse was 160 m × 88 m in length and width and had 11 spans with two roofs in total with the greenhouse ridge facing the north–south direction. The greenhouse is covered with 4+9A+4 mm double-layer hollow tempered glass, and the tempered glass is buttressed with 0.5 mm inner white and outer gray corrugated seamless butt plate, and the external white rubber seal is used. The roof and windows in the roof are covered with 4 mm scattering tempered glass and 0.6 mm white seamless steel plate. The opening ventilation windows were located on the roof with an internal shade and electric side insulation system. The entire greenhouse will be completely closed when the windows are closed, and show the characteristics of semi-closed buildings when the windows are opened. The greenhouse environment was equipped with an irrigation, heating, CO_2_ supplemental gas, planting, harvesting, light supplemental, fertilization, and automatic control systems, and other environmental control equipment. No cooling equipment was installed and the temperature conditioning relied on the mist spray system. The planting area in the greenhouse was divided into two blocks and substrate-cultivated tomatoes were planted in these two blocks, as shown in Fig. 1.

**Fig. 2:**
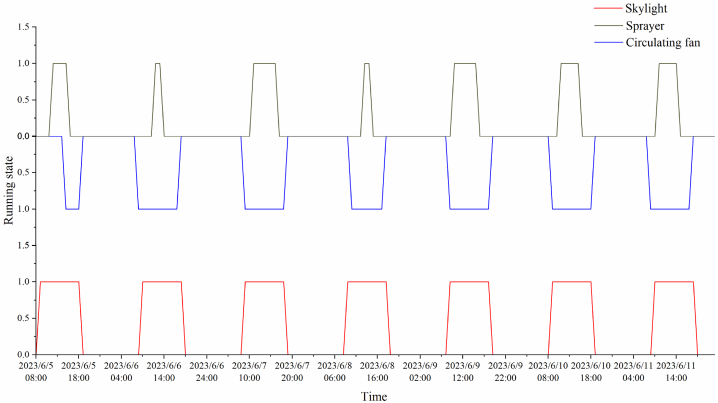
Running state of greenhouse cooling system.

In the greenhouse, each system was operated by staff and the Hoogendoor system (one of the front runners in the horticultural industry). Each morning at approximately 08:00 h, the staff activated the shed operation, greenhouse control system, sensor system to measure greenhouse temperature and humidity, carbon dioxide, and light measurements as well as automatic control of the greenhouse, such as opening the lee window, circulation fan and fresh air unit, and greenhouse temperature control and dehumidification. Greenhouses are divided into southern and northern regions, which are divided into a number of districts, and manual operation is based on more detailed regulations for different regions of the environment. During the whole test period, the roof windows of the greenhouse, the mist spray system and the circulation fan system remained open to condition indoor air temperature as shown in Fig. 1(b). And the opening area of the skylight, humidification and circulation fan was jointly controlled manually and their operating status is shown in Fig. 2. The greenhouse was closed at 18:00 h, and the staff activated the silent state environmental control system which performed necessary regulation in extreme cases.

### 2.2 Experimental protocol

**Fig. 3:**
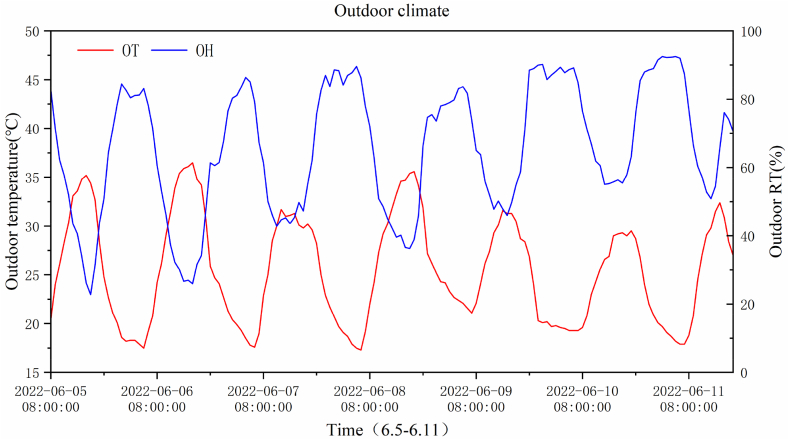
Outdoor temperature and humidity.

The experiments were conducted from June 5–11, 2022, and data from 08:00–18:00 h in typical summer weather were selected for analysis. Considering the layout of the crops grown in the greenhouse, the sensors were arranged according to Fig. 2. The weather conditions from June 5–11 are listed in Table 1. Outdoor temperature and humidity collected by weather stations are shown in Fig. 3.

**Table 1:** Outdoor air temperature during experiments.

Date	Maximum temperature	Minimum temperature	Weather
June 5, 2022	31 °C	14 °C	Cloudy
June 6, 2022	33 °C	16 °C	Sunny
June 7, 2022	31 °C	17 °C	Cloudy
June 8, 2022	34 °C	19 °C	Cloudy
June 9, 2022	30 °C	16 °C	Cloudy
June 10, 2022	31 °C	19 °C	Cloudy
June 11, 2022	32 °C	20 °C	Moderate rain

**Fig. 4:**
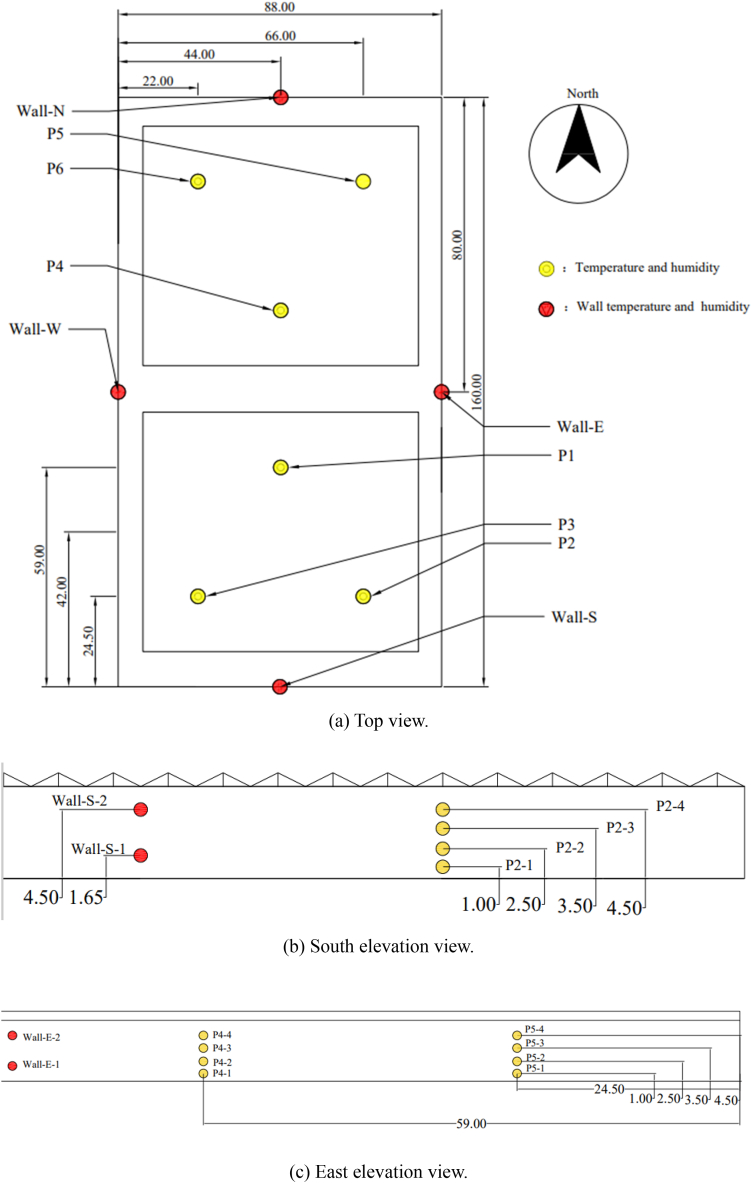
The measurement point arrangement.

As shown in Fig. 4(a)-4(c), six sites in the horizontal direction were selected in the greenhouse for temperature and humidity measurements. At each site, four temperature and humidity sensors were installed in the crop roots, middle layer, canopy layer and 1.5 m above the canopy layer, respectively. The temperature and humidity data were collected by the sensors once every 5 min. The specifications of these sensors are listed in Table 2.

**Table 2:** Measurement devices.

Measured parameters	Instrument	Model	Measuring range	Measurement accuracy
Temperature and humidity	Temperature and humidity recorder	COS-04	−40∼+80 °C 0∼100%RH	±0.3 °C（25 °C） ±2%RH（60 %, 25 °C）

As the north and east sides of the greenhouse are neighbors to other indoor zones, and the south and west sides are adjacent to outdoor air, it is necessary to record the differences in different boundaries. A total of eight sensors arranged in two layers, one at the middle height of the cultivation area and one at 1.5 m above the canopy, were placed on the four sidewalls of the greenhouses, as shown in Fig. 4 (a)–(c), and the same settings as those in the air were set to collect data once every 5 min.

In addition to temperature, humidity was also monitored. Six points were arranged in the greenhouse for wind speed and direction measurements, and each point was divided into two layers, as shown in Fig. 4(c), which were the middle layer of the crop and 1.5 m above the canopy, and handheld sensors were used for measurement. During the measurement period, after the instrument indicated a stable number, a reading was taken every 5 s, and the average value of five measurements was obtained for each measurement. The outdoor environmental parameters, including temperature, humidity, light, wind speed, and CO_2_ concentration, were measured by a weather station installed outdoors, which was placed in an open area 20 m northwest of the greenhouse.

## 3 Results and discussion

### 3.1 Greenhouse temperature and humidity

**Fig. 5:**
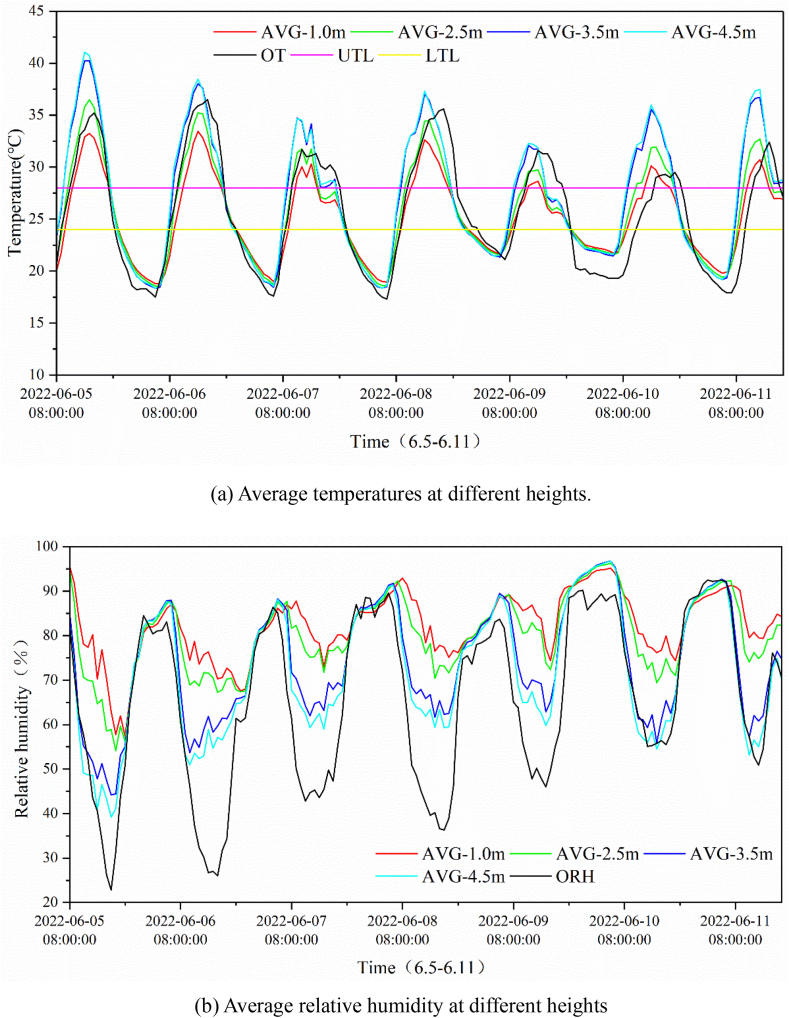
Average temperature and humidity in the greenhouse. AVG-1.0 m, AVG-2.5 m, AVG-3.5 m and AVG-4.5 m are the average values at the measurement points with the heights of 1.0 m, 2.5 m, 3.5 m and 4.5 m, respectively; OT and ORH are the temperature and relative humidity of ambient environment, respectively; UTL and LTL are the upper and lower temperature limits suitable for tomato crop growth.

Fig. 5(a) and (b) show a comparison between the average temperature and humidity respectively inside and outside the greenhouse from June 5–11. It can be seen that the temperature inside the greenhouse fell below the lower limit of the tomato demand temperature (LTL) from 08:00 h. All the temperatures inside the greenhouse rose gradually as the sun rose and exceeded the upper limit of tomato cultivation demand temperature (UTL) after 11:00 h, reaching a peak at approximately 14:00 h. As the sun set, the temperature inside the greenhouse began to drop until approximately 21:00 h, after which the temperature declined below the LTL of tomato cultivation requirements. The variations in temperature and humidity inside the greenhouse showed some displacement to those outdoors, that is, the temperature and humidity inside the greenhouse always dropped or rose in advance of the outdoor temperature and humidity changes. This may be because of the effects of solar radiation. Greenhouses tend to transfer solar radiation energy into sensible air temperatures. The variation in relative humidity inside the greenhouse contrasted to that of temperature because the relative humidity is related to temperature, and higher temperatures lead to lower relative humidity for the same moisture content. After sunrise, there was stratification of both temperature and humidity inside the greenhouse, with a small difference in temperature and humidity between the 4.5 m and upper layers, the middle and lower layers, and a large difference in temperature and humidity between the 4.5 m and upper layers, and the middle and lower layers, with the maximum temperature and humidity difference occurring at 12:00 h. After sunset, the temperature and humidity inside and outside the greenhouse remained constant. During the test period, the temperature difference at different points in the greenhouse varied from 0 to 20 °C, while the humidity difference varied from 0 to 45 %. The difference between average indoor and outdoor temperatures ranged from -5–10 °C. The humidity fluctuation in the lower and middle layers of the greenhouse was minimal, except for June 5, which was 0–20 %. There was a substantial difference between the outdoor and indoor humidity in the 4.5 m and upper layers in the greenhouse from June 5–9, while there was a smaller difference between June 10–11.

After the operational activation of the greenhouse, the indoor environmental control system, including the spraying system, circulating fan, and skylight, was activated, so that the temperature inside the greenhouse increased and humidity decreased, reaching the maximum/minimum value at around 14:00 h. Subsequently, the outdoor temperature decreased, and humidity increased, displaying the same trend in the greenhouse. This indicates that after 14:00 h, the environment inside the greenhouse was influenced more by outdoor factors. The changes in the temperature and humidity in the middle and lower indoor layers were minimal, and there was a distinct stratification phenomenon between the upper and lower layers. The stratification may be attributed to the blocking effect of crops on light and airflow. During the data measurement, there was no obvious difference in the temperature and humidity variation under different weather conditions, which indicates that the weather has little effect on the indoor environment in the greenhouse.

### 3.2 Wall temperature and humidity

**Fig. 6:**
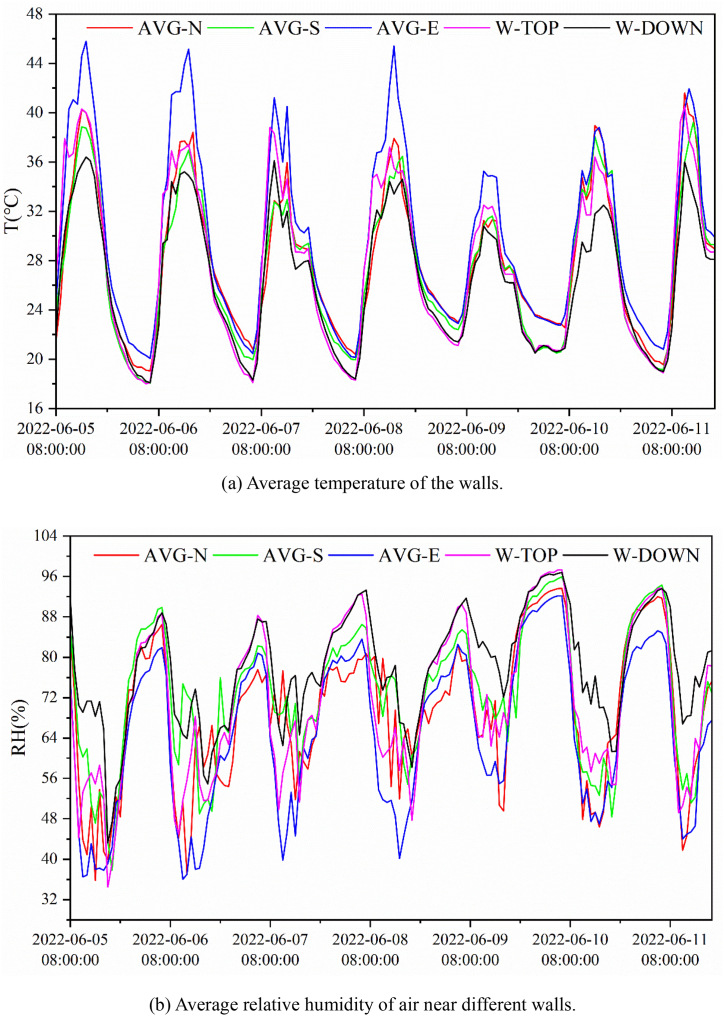
Greenhouse wall temperature and humidity. AVG-N, AVG-S, AVG-E are the average values of temperature and relative humidity on the north, south and east walls respectively; W-TOP and W-DOWN are the average values on upper and lower parts of the west wall.

Fig. 6(a) and (b) show the temperature and humidity of the greenhouse walls over time respectively. As there is no obvious difference in the temperature and humidity in the upper and lower parts of the walls except for the west wall, only the average values are shown, while the temperature and humidity in the upper and lower parts of the west wall were determined. It can be seen from Fig. 6 (a) and (b) that the variations in the wall temperature and humidity over time were consistent with the air temperature and humidity in the greenhouse. During the measurement period, the temperature and humidity on the east wall showed the greatest variation compared to the other walls, possibly because of the longest exposure to sunlight. Meanwhile, the stratification of temperature and humidity in the upper and lower parts of the west wall was evident.

### 3.3 Non-uniform characteristics of greenhouse environmental parameters

Based on the measured data, non-uniform coefficients were calculated to characterize the non-uniformity of the indoor environment in the greenhouse according to Eq. (1) [33]:

(1) $$X=\frac{\sqrt{\frac{1}{n}\Sigma \left(C_{i}-\bar{C}\right)}}{\bar{C}}$$

where *X* is the non-uniform coefficient, smaller *X* indicates better homogeneity, *n* is the number of measurement points, $C_{i}$ and $\bar{C}$ are the environmental parameters and their averages, respectively.

**Fig. 7:**
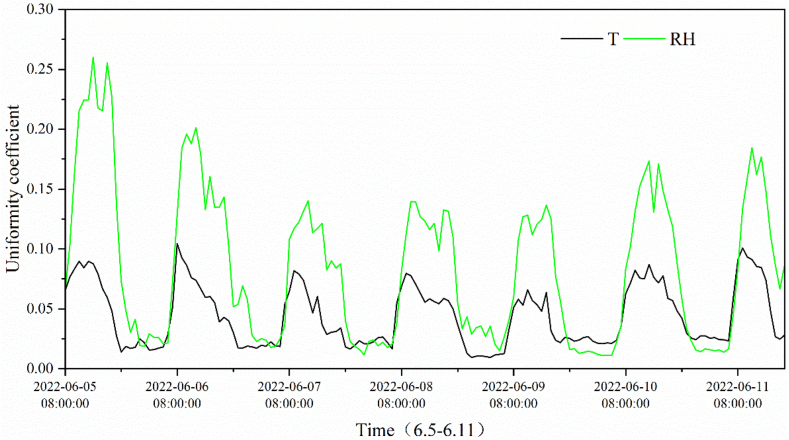
The overall spatial non-uniform coefficients over time.

Fig. 7 shows the overall spatial non-uniformity coefficients of temperature and humidity over time in the greenhouse. It can be seen that they vary periodically over time, in accordance with the normal distribution, and the non-uniformity coefficients of humidity appear greater than those of temperature. A similar trend was revealed between the non-uniform variation of temperature and humidity, and there was a delay of 1–2 h between them. This may be because the humidity in the greenhouse was more sensitive to equipment conditioning owing to the dual effect of temperature and moisture on relative humidity. During the measurement period, the overall non-uniform coefficients of humidity were higher on June 5 and 6, up to 0.25 and 0.20, respectively, while those of the temperature appeared steadier in the range of 0 and 0.1, respectively.

**Fig. 8:**
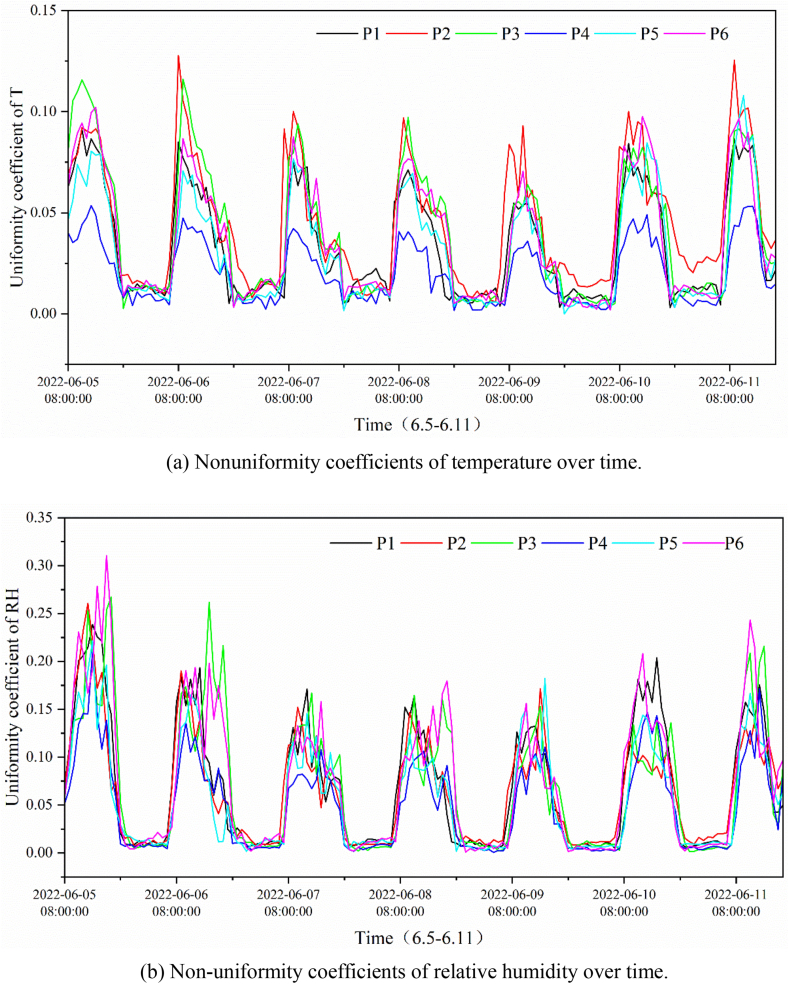
The non-uniform coefficient through the vertical direction at different points in the greenhouse.

Fig. 8(a) and (b) show the variation in the nonuniform coefficient of air temperature and relative humidity in the vertical direction at different points in the greenhouse. The non-uniformity of temperature and humidity showed a similar variation over time. During opening time, the non-uniformity coefficients of temperature and humidity in the greenhouse ranged from 0.05 to 0.125, and from 0.05 to 0.275, respectively. The results indicated that during the opening period, non-uniformity of both temperature and humidity existed in the height direction in the greenhouse, and humidity was more uneven than temperature. After 18:00 h, the nonuniformity coefficient was close to zero, and the environment in the greenhouse was more uniform. The main cause of this may be the effect of solar radiation. Most of the differences in the non-uniform coefficients of temperature and humidity at different points in the greenhouse were in the range of 0–0.125 and 0–0.30 respectively.

**Fig. 9:**
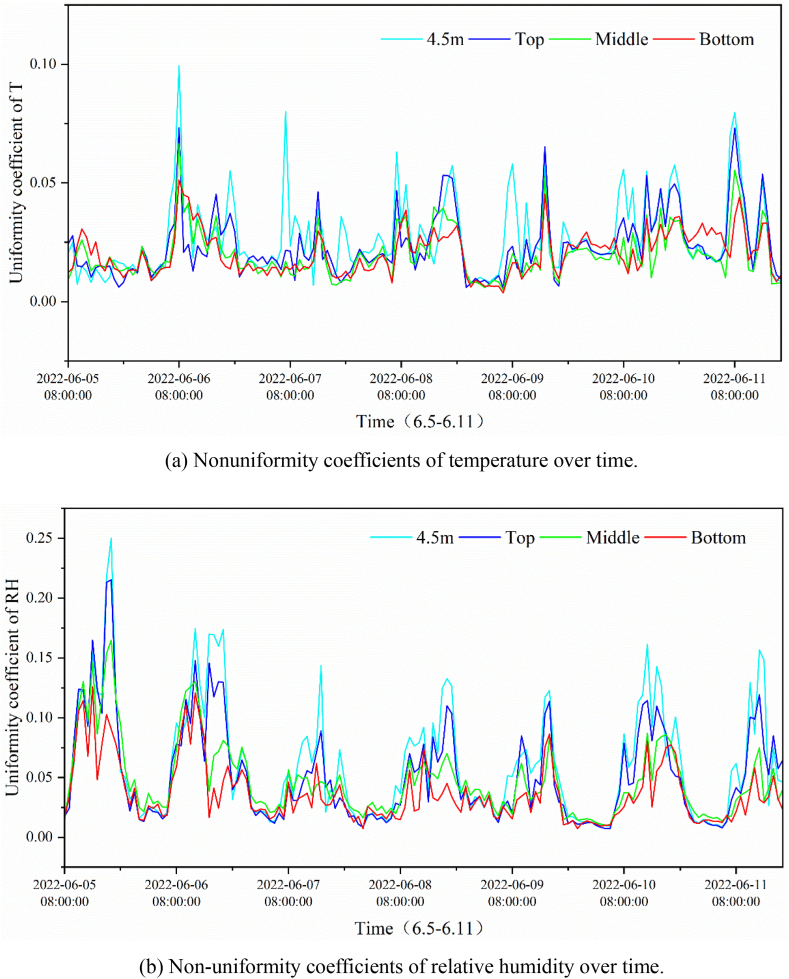
The non-uniform coefficient through the horizonal direction at different points in the greenhouse.

Fig. 9(a) and (b) show the non-uniform coefficients of temperature and humidity respectively in the horizontal direction at different heights in the greenhouse. During the opening period, although highly random characteristics of the non-uniform coefficient of temperature and humidity were shown, the non-uniformity coefficients of temperature and humidity at a height of 4.5 m and the upper part were clearly greater than those at the middle and lower levels most of the time. During the measurement period, the non-uniformity coefficient of temperature was low on June 5, lying between 0 and 0.025, while the non-uniformity coefficient of humidity was high, with a maximum value of 0.25.

Comparing Fig. 5, Fig. 7, the non-uniformity of the temperature and humidity in the vertical direction in the greenhouse was more remarkable than that in the horizontal direction. This may be because of the shading and buffering effects of the crop. The non-uniformity coefficient at the same horizontal level does not conform to the normal distribution because of the uneven water distribution of the spraying system in the greenhouse and the mixing of the air by circulating fan. After encountering water spraying in a certain position, the temperature drops and the humidity rises. The air was then stirred and mixed using a circulating fan. In this case, the temperature and humidity of the air are regulated to some extent, resulting in fluctuations in temperature and humidity over short periods of time. It was also found that the non-uniform characteristics of temperature and humidity differed with weather conditions, which clearly contrasts on June 5 (cloudy day) and 6 (sunny day). In summary, the uneven temperature and humidity environment was generated primarily by the environmental control system, buffering and shading effect of crops, and weather conditions.

### 3.4 Theoretical analysis on the potential of energy saving using non-uniform environmental conditioning

With the assumption that only sensible heat is extracted from the greenhouse for simplification, the energy consumption for environmental conditioning to the temperature and humidity required by the crops can be estimated based on the measured actual temperature in the greenhouse using Eq. (2).

(2) $$Q=\rho Vc_{p}\left(T_{actual}-T_{desired}\right)$$

where, *Q* is the required cooling or heating energy, kJ, a negative value represents heating and a positive value represents cooling; *ρ* is the density of air, 1.29 kg/m^3^; *V* is the volume of the space requiring environmental conditioning; *c*_*p*_ is the specific heat capacity of air, 1.01 kJ/(kg•K), *T*_actual_ is the measured temperature over time in different layer zones in the greenhouse, K; *T*_desired_ is the corresponding required temperature for the growth period of the crop, In this study, tomato in the fruiting period was the cultivated crop and the desired temperature range was 24–28 °C. Hence, 28 °C was set as the desired cooling temperature and 24 °C was set as the desired heating temperature. During this period, the total energy consumption should be the sum of the heating and cooling energy consumption.

Based on Eq. (2), the energy consumption of the two conditioning modes, including overall and zone-specific conditioning was calculated. In the overall conditioning mode, the air temperature in the entire space in the greenhouse was conditioned, whereas in the zone-specific conditioning mode, only the air temperature that the crops occupy was conditioned, which is a non-uniform conditioning mode. For simplification, the greenhouse was divided into three parts: the cultivation, above cultivation, and below cultivation areas, as listed in Table 3. The cultivation area consisted of three parts: the root, middle, and canopy layers, and the small difference in the temperature requirement was ignored. Considering the more uniform environment in the horizontal direction, as mentioned in Section 3.3, the space in the horizontal direction was not further divided. Thus, the energy consumption of the overall conditioning mode can be estimated using Eq. (3) and that for the zone-specific conditioning mode can be estimated using Eq. (4).

(3) $$Q_{overall}=Q_{Top\;layer}+Q_{cultivated\;area}+Q_{Bottom\;layer}$$

(4) $$Q_{zone-specific}={Q_{cultivated\;area}=Q}_{canopy}+Q_{middle}+Q_{root}$$

**Table 3:** Sub-zone division in the greenhouse.

		Length/m	Width/m	Height/m	Volume/m³
Top layer		88	67	3.2	37,734
Cultivated layer	Total	88	67	2.5	29,480
	Canopy	88	67	0.75	8844
	Middle	88	67	1.0	11,792
	Root	88	67	0.75	8844
Bottom layer		88	67	1.0	11,792

**Fig. 10:**
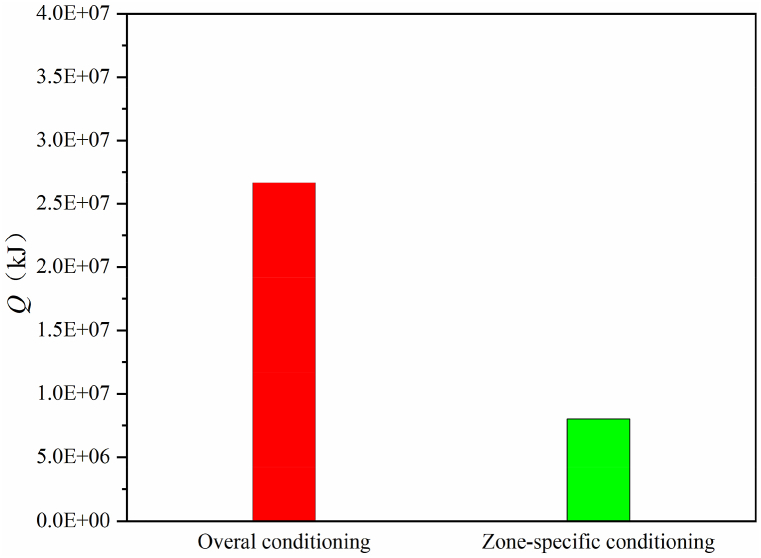
Estimated energy consumption for heating or cooling by overall and zone-specific conditioning.

Using Eqs. (2)–(4), the total energy consumption for the two conditioning modes in the measured period was calculated when conditioning was limited from 08:00 h to 18:00 h. The results are shown in Fig. 10. Clearly, a large amount of energy (up to 69.9 %) can be saved when the zone-specific conditioning mode alters the common overall conditioning mode. This can be achieved because the top zones of the greenhouse without occupation of crops are not conditioned by the zone-specific conditioning, where the air temperature is the highest with large temperature differences of up to more than 5 °C compared with that in the bottom zones, as shown in Fig. 5. The rough estimation results indicated the promising energy-saving potential of non-uniform environmental conditioning applied in greenhouses.

## 4 Conclusions

In this study, by exploring the stratification phenomena of environmental parameters, including temperature and relative humidity inside the greenhouse and analyzing the non-uniform variation of the greenhouse field environment, it was found that the trends of temperature and relative humidity changes in the greenhouse were contrasting, and relative humidity often decreased when the temperature rose. Compared with the high layer at a height of 4.5 m in the greenhouse, the middle and lower layers of the cultivation area displayed small and stable fluctuations in temperature and humidity, whereas the upper layer of the cultivation area displayed a similar trend of temperature and humidity changes as that of the higher layer at a height of 4.5 m. The cloudy weather conditions had little effect on the temperature and relative humidity in the greenhouse but affected the greenhouse walls more significantly. The east wall of the greenhouse displayed the largest temperature and humidity variation due to the longest exposure to sunlight, and the stratification of temperature and humidity on the west wall was more distinct due to shadows created by crops, while the temperature and humidity of the other walls were consistent.

After analysis of measured temperature and humidity, it was found that the non-uniformity in summer in the vertical direction was larger than that in the horizontal direction. This may be caused by the uneven regulation of the environmental control equipment in the greenhouse and buffer shading by the crop. Based on this phenomenon, cultivation zone-specific environmental conditioning was proposed in concept, and the energy-saving potential for the conditioning mode was estimated and compared with that of the overall environmental conditioning. It was found that the energy consumption for the measurement period could be reduced by 69.9 % compared with that of the traditional environmental conditioning mode. Thus, it can be preliminarily concluded that zone-specific environmental conditioning is a promising energy-efficient technique for application in greenhouses. Further research on technology development details of the principle of non-uniform thermal environmental formation is necessary. In addition, in contrast to indoor environmental conditioning in residential buildings, CO_2_ and crop growth are two more unique and important issue in greenhouses, however, they were not investigated in our study, resulting in short measurement time and less data volume. These issues need to be further considered in our further research to increase crop production.

## Funding statement

This research was financially supported by the Shaanxi Key Research and Development Program, China (2022ZDLNY03-02), Shaanxi Provincial Technology Innovation Guidance Special Fund, China (2021QFY08-02), and the Scientific & Technological Innovative Research Team of Shaanxi Province, China.

## Data availability statement

Data included in article/supp. Material/referenced the in article.

## CRediT authorship contribution statement

**Xianpeng Sun:** Writing – original draft, Validation, Project administration, Investigation, Formal analysis, Data curation. **Jinhong He:** Writing – original draft, Validation, Software, Formal analysis. **Chuanzhen Li:** Writing – original draft, Resources, Investigation, Data curation. **Yangda Chen:** Project administration, Methodology, Formal analysis, Data curation. **Runjie Li:** Writing – review & editing, Writing – original draft, Resources, Methodology. **Ziteng Wang:** Supervision, Resources, Methodology. **Weijun Wu:** Project administration, Methodology, Investigation. **Yapeng Li:** Supervision, Software, Conceptualization. **Xuxin Guo:** Visualization, Software, Resources. **Xinke Wang:** Writing – review & editing, Resources, Funding acquisition, Conceptualization.

## Declaration of competing interest

The authors declare that they have no known competing financial interests or personal relationships that could have appeared to influence the work reported in this paper.
